# Discrimination Experiences during COVID-19 among a National, Multi-Lingual, Community-Based Sample of Asian Americans and Pacific Islanders: COMPASS Findings

**DOI:** 10.3390/ijerph19020924

**Published:** 2022-01-14

**Authors:** Van M. Ta Park, Marcelle M. Dougan, Oanh L. Meyer, Bora Nam, Marian Tzuang, Linda G. Park, Quyen Vuong, Joon Bang, Janice Y. Tsoh

**Affiliations:** 1 Department of Community Health Systems, School of Nursing, University of California—San Francisco, San Francisco, CA 94143, USA; bora.nam@ucsf.edu (B.N.); yuan.tzuang@ucsf.edu (M.T.); Linda.Park@ucsf.edu (L.G.P.); 2 Asian American Research Center on Health (ARCH), University of California—San Francisco, San Francisco, CA 94143, USA; janice.tsoh@ucsf.edu; 3 Department of Public Health and Recreation, San Jose State University, San Jose, CA 95192, USA; marcelle.dougan@sjsu.edu; 4 Department of Neurology, School of Medicine, University of California, Davis (UCD), Sacramento, CA 95817, USA; olmeyer@ucdavis.edu; 5 International Children Assistance Network (ICAN), 532 Valley Way, Milpitas, CA 95035, USA; quyen.vuong@ican2.org; 6 Iona Senior Services, 4125 Albemarle Street NW, Washington, DC 20015, USA; jbang@iona.org; 7 Department of Psychiatry and Behavioral Sciences, School of Medicine, University of California—San Francisco, San Francisco, CA 94143, USA

**Keywords:** COVID-19, discrimination, Asian Americans, native Hawaiians and Pacific islanders

## Abstract

Reports of escalated discrimination among Asian Americans and Pacific Islanders (AAPIs) due to COVID-19 are alarming, making this a public health priority. However, there are limited empirical studies on the scope and impact of COVID-19-related discrimination among AAPIs. Using the COVID-19 Effects on the Mental and Physical Health of AAPI Survey Study (COMPASS) data (N = 4971; survey period: October 2020–February 2021), which is a U.S.-wide multi-lingual survey, we examined the prevalence of, and factors associated with discrimination experiences attributable to being an AAPI during the COVID-19 pandemic. Overall, 60.7% reported experiencing discrimination; the group prevalence ranged from 80.0% (Hmong) to 40.5% (Native Hawaiians and Pacific Islanders). Multivariable logistic regression models revealed that COVID-19-related factors were associated with many discrimination experiences: having a shelter-in-place order of ≥1 month, living in areas with perceived similar/higher COVID-19 severity, and negative impact in family income/employment due to COVID-19. Additionally, being Asian American (versus Native Hawaiians and Pacific Islanders), females, non-heterosexuals, younger, more severe effect on family income, living in the non-West, and poorer health were significantly correlated with discrimination experiences. Findings may assist in formulating anti-AAPI-discrimination policies and programs at the local, state, and federal levels. Culturally appropriate programs and policies to combat this are urgently needed.

## 1. Introduction

Reports of escalated discrimination among Asians and people of Asian descent worldwide [1] and in the United States [2] fueled by racist and xenophobic rhetoric related to COVID-19 are alarming, making this a public health priority. Focusing on the United States, although individual reports of discrimination, which is defined as “the unfair or prejudicial treatment of people and groups based on characteristics such as race, gender, age or sexual orientation” [3], have surfaced, the scope and impact of COVID-19-related discrimination/xenophobia among Asian Americans and Pacific Islanders (AAPIs) have not been empirically studied very much. The sparse data that exist suggest that AAPIs experience high rates of COVID-19-related discrimination.

Pre-COVID-19, 13–50% of Asian Americans (ASAs) reported discriminatory experiences associated with race across different settings (e.g., healthcare, employment, housing) [4,5,6,7]. In California, AAPIs were more likely than other Californians in general to have experienced workplace racial discrimination and the rates of racial discriminatory experience differed by AAPI subgroups [8]. Evidence has shown a consistent link between discrimination experiences and adverse mental-health outcomes [4,9,10]. As a result of racial discrimination, ASAs (vs. Whites) were more likely to avoid seeking health care and accessing housing [4].

Since the COVID-19 outbreak, ASAs have been victims of social stigma, racist incidents, and hate crimes related to COVID-19 [11,12]. A poll found 32% of the adult U.S. respondents have witnessed Asians being blamed for the coronavirus epidemic, and among Asian respondents, 60% have witnessed the same problem [13]. The “Stop AAPI Hate” website received nearly 1500 reports of COVID-19-related discrimination against ASAs within the first four weeks despite shelter-in-place (SIP) orders having been implemented across many parts of the country [14]. Between March 19, 2020 and June 30, 2021, incident reports were received from all 50 states and the District of Columbia, with 54.6% of the reports from California and New York. Race was cited as the primary reason for discrimination (90%) [2].

COVID-19-related discrimination was not limited to large Asian subgroups, such as Chinese Americans. Indeed, 56.5% of the hate incident reports made to Stop AAPI Hate were by non-Chinese ASAs and among these, 16.8% were reported by Koreans, 9.1% by Filipinos, and 8.6% by Japanese. The most frequent locations of discrimination were in public streets (31.6%), at businesses (30.1%), 9.4% in private residence, and 8.8% were online. Ethnic Chinese became targets, exemplified by the fact that 48.1% of the hate incident reports contained at least one xenophobic statement (e.g., anti-China).

Given the rise of COVID-19-related hate among AAPIs and the diversity of AAPIs [15], this study used data from a national, multi-lingual, community-based survey called COVID-19 Effects on the Mental and Physical Health of AAPI Survey Study (COMPASS) to examine the proportion of and factors associated with discrimination experiences during the COVID-19 pandemic due to being AAPIs.

## 2. Materials and Methods

### 2.1. Study Eligibility, Recruitment and Procedures

COMPASS is a community-based national survey that assesses the COVID-19 effects on AAPIs. Eligible participants must self-identify as AAPI alone or in combination with other races/ethnicities, must be able to read English/Chinese (Traditional/Simplified Chinese)/Korean/Samoan/Vietnamese, must be ≥18 years old, and must reside in the U.S. COMPASS was translated into the above-mentioned languages given the high proportions of limited English proficiency (LEP) among Vietnamese (50%), Koreans (46%) and Chinese (40%) in the U.S. [16] as well as to encourage greater participation from Samoans (16% with LEP) [17]. The survey could be completed online via study website (https://compass.ucsf.edu), over the phone or in-person. The survey completion rate was 79.4%.

This study reports on the 4971 participants who completed the survey from October 2020–February 2021. Most of them (89%) completed the survey by themselves and the remaining received assistance from family/friends/staff. The mean survey-completion time was 21.4 min (standard deviation (SD) = 15.1). Each participant was provided an option of receiving a $10 gift card upon survey completion. The survey used Research Electronic Data Capture (REDCap) tools hosted at University of California San Francisco [18,19].

Participants heard about COMPASS through community partners who serve AAPIs, personal/professional networks, social media, email/listservs, flyers, and ethnic media. COMPASS also recruited participants from the Collaborative Approach for AAPIs Research and Education (CARE) Registry [20].

The World Health Organization’s process of translation and adaptation of instruments [21] was used to guide the translations of the study materials that were not already available in the targeted language(s).

### 2.2. Measurement Framework

We applied the COMPASS measurement framework of discrimination experiences during COVID-19 pandemic. This framework, guided by the previously discussed literature and the National Institute of Minority Health and Health Disparities (NIMHD) Research Framework [22], examined the multi-level influences on discrimination experiences, which operate within the sociodemographic contexts of AAPIs (e.g., cultural group, age, sex, sexual orientation, education, income, English proficiency, nativity, years in the US) that cut across individual (general health, COVID-19 status), interpersonal (marital status, caregiver status), community (geographic regions, perceived severity of COVID-19), and societal (length of shelter-in-place) levels that are modifiable and COVID-specific.

### 2.3. Measures

#### 2.3.1. Discrimination Experiences during COVID-19 Pandemic

Perceived discriminatory experience was assessed by using a revised 8-item Everyday Discrimination Scale (EDS) [6,23]. Participants were asked, “In the past 6 months, how often have…” with each of the 8 items ending with “because you are Asian, Asian American or Pacific Islander.” The 8 items were:

1. you been treated with less respect than other people…
2. you been treated unfairly at restaurants or stores…
3. people criticized your accent or the way you speak…
4. people acted as if they think you are not smart…
5. people acted as if they are afraid of you…
6. people acted as if they think you are dishonest…
7. people acted as if they’re better than you are…
8. you been threatened or harassed…

We used in “the past 6 months” for COMPASS (original EDS used “12 months”) to measure the occurrence of discrimination experienced within the duration of the COVID-19 pandemic using a 4-point scale: 0 (never), 1 (rarely), 2 (sometimes), and 3 (often). The standardized Cronbach alpha for the EDS in this study was 0.93. The EDS is widely used with high reliability [6,24,25] and construct validity [26,27], and has been studied in different racial/ethnic groups, including ASAs, and is reported to have adequate psychometric properties [6,28,29,30,31,32]. However, we acknowledge that these studies have primarily focused on certain AAPIs (e.g., Chinese, Vietnamese), and have not been sufficiently validated with other AAPI groups (e.g., South Asians, NHPIs).

#### 2.3.2. Sociodemographic Characteristics

The participant’s demographic information was drawn from CARE’s Sociodemographic Survey [20], and thus, readily translated into all target languages. Participants were asked about their race, ethnic/cultural group, sex, sexual orientation, year of birth, nativity, years lived in the U.S., employment, education, and annual household income in 2019. The participant had LEP if they indicated that they can speak, read, and/or write English “a little bit” or “not at all”.

#### 2.3.3. Individual Level Characteristics

General health was measured by asking about participants’ health “today” on a scale from 0 (worst) to 100 (the best health you can imagine) using the EQ-5D [33,34] item, which was categorized into quintiles.

COVID-19 status was measured by one item from the Questionnaire for Assessing the Impact of the COVID-19 Pandemic and the accompanying mitigation efforts on Older Adults (QAICPOA) [35]. The participants were asked, “Have you been diagnosed with COVID-19 by a doctor or other health care provider?” Response options included: yes; no; and, unsure.

#### 2.3.4. Interpersonal Level Characteristics

Marital status and caregiving status were again drawn from CARE’s Sociodemographic Survey [20]. The participants were asked whether they are caring for minor children, older adults, or individuals with special needs (Yes/No).

#### 2.3.5. Community Level Characteristics

U.S. geographic region was obtained per the Census Bureau’s definition of region (Midwest/Northeast/South/West) [36] by converting the zip code, or internet protocol address in the case of missing zip codes (n = 146).

Perceived Severity of COVID-19 was developed by COMPASS. Participants were asked, “How would you rate the severity of the COVID-19 outbreak where you live in comparison to other locations in the US?” Responses were recorded on a 5-point Likert scale (1 = a lot less severe than most other places in the U.S., 2 = somewhat less severe, 3 = about the same, 4 = somewhat more severe, 5 = a lot more severe).

#### 2.3.6. Societal Level Characteristics

The length of shelter-in-place (SIP) was developed by COMPASS and asked, “How long was the shelter-in-place (or stay-at-home) order at where you live?” Responses included 0 (no order); 1 (<1 month), 2 (1–2 months), and 3 (≥2 months).

### 2.4. Statistical Analysis

The perceived discrimination (measured by EDS) weighted the response options based on situation-based coding [37]. In other words, the study’s outcome was modeled as a dichotomous variable: participants who responded “never” to all items were categorized as having no discrimination experience, and those who experienced any discrimination (i.e., “rarely/sometimes/often” to any of the EDS items) were categorized as having “any” discrimination experience. The categories were classified as such because we were interested in examining any experiences of discrimination, and even if a person considers their experience rare, it is still important to capture. In some cases, a ‘rare’ experience of discrimination may reflect a lower level of engagement with the public due to being largely confined in the home because of SIP and/or fear of anti-AAPI hate; however, these experiences, when they do occur, are still important to capture and not minimized in their importance. Previous research had also dichotomized the EDS similarly [38,39].

For descriptive statistics, this study used chi-squared tests to examine the relationships between sociodemographic variables and perceived discrimination and modeled the odds of experiencing discrimination using multivariable logistic regressions. The reference groups for categorical variables were selected to identify the predictors of increased odds of discrimination experiences. For the cultural group, Asian Indian was selected as the reference group since they reported the lowest proportion of any discrimination experiences among all ASAs. In post-hoc analyses, comparisons between cultural group were conducted. Any variable that had a *p*-value of <0.10 in the bivariate analyses with the outcome was a candidate for inclusion in the final model. Nativity and LEP were correlated in our population. Therefore, we selected LEP based on the lower AIC of the model. Further, because change in family income/employment and income were also correlated, with those at lower income levels more likely to experience changes to their family income/employment, change in family income/employment was selected for the final model based on the lower AIC. The Receiver Operating Curve was used to examine how well the variables in the model predicted the outcome. The area under the curve was 0.7, suggesting an acceptable predictive model. All statistical tests were two-sided.

## 3. Results

### 3.1. Sample Characteristics

A total of 4971 participants completed the survey (Table 1). The mean age was 45.2 years (SD = 16.4). The major cultural groups included ethnic Chinese (including persons from China, Hong Kong, and Taiwan; (33.9%), Korean (22.5%), and Vietnamese (19.3%). Many were females (64.1%), foreign-born (63.4%), and married/living with a partner (64.8%). Most identified as heterosexuals (91.2%). Many resided in the western U.S. region (64.8%), 22% had LEP, and 24% were caregivers.

### 3.2. COVID-19-Related Discrimination Experience

Overall, 60.7% reported one or more of the nine discrimination experiences during the COVID-19 pandemic; prevalence by group were 80.0% Hmong, 64.7% ethnic Chinese, 64.2% Korean, 61.3% Filipino, 57.7% Japanese, 55.7% Vietnamese, 41.5% Asian Indian, and 40.5% NHPI (Table 2). The mean number of different types of discrimination experiences is 2.59 (2.86 SD; range 0–8). Table 2 also shows varying descriptions for each EDS item by cultural groups (e.g., 42.7% of Hmong indicated experience with, “people acted as if they think you are dishonest” or that “you have been treated unfairly in restaurants/stores”; 41.8% of ethnic Chinese indicated experience with “people acted as if they are afraid of you”).

**Figure 1:**
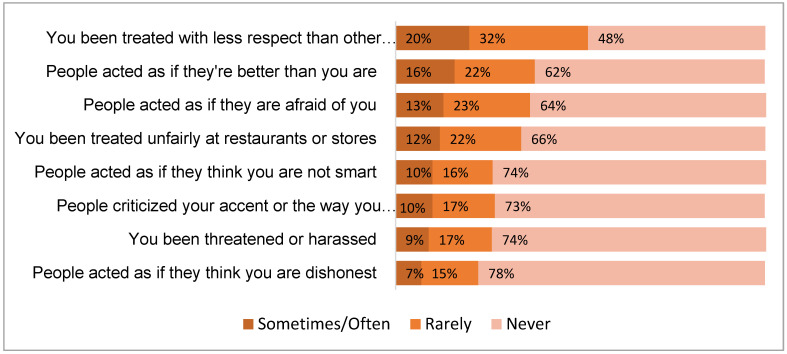
Experiences of discrimination during the COVID-19 pandemic due to being Asian, Asian American or Pacific Islander.

Figure 1 shows the proportions by EDS item, which ranged from 22% who said “rarely,” “sometimes,” or “often” to the item, “People acted as if they think you are dishonest because you are AAPI,” to 52% for the item, “You have been treated with less respect than other people because you are AAPI”.

### 3.3. Bivarate and Multivariable Analyses

In bivariate analyses (Table 3), cultural group, sex, sexual orientation, age, nativity, LEP, marital status, employment status, education, income, length of SIP orders, perceived severity of COVID-19, change in family income/employment as a result of COVID-19, region, and self-reported health status were significantly associated with everyday discrimination experiences. However, there was not a significant association of caregiver status.

In the adjusted model (Table 3), the associations of cultural group, sex, sexual orientation, age, income, length of SIP orders, perceived severity of COVID-19, change in family income/employment as a result of COVID-19, region, and self-reported health status persisted. Compared to Asian Indian, ethnic Chinese (adjusted odds ratio (aOR) = 2.38 [95% CI: 1.79–3.17]), Filipino (aOR = 1.81 [95% CI: 1.18–2.78]), Hmong (aOR = 2.09 [95% CI: 1.10–3.97]), Japanese (aOR = 2.21 [95% CI: 1.49–3.28]), Korean (aOR = 2.04 [95% CI: 1.50–2.78]), Vietnamese (aOR = 1.61 [95% CI: 1.18–2.20]), and other/mixed (aOR = 1.78 [95% CI: 1.23–2.57]) were significantly more likely to report everyday experiences of discrimination. In post-hoc analyses, ethnic Chinese, Japanese, and Korean were significantly more likely to report everyday experiences of discrimination compared to NHPI: aOR = 2.25 [95% CI: 1.45–3.50], aOR = 2.09 [95% CI: 1.25–3.50] and 1.93 [95% CI: 1.22–3.05], respectively. Ethnic Chinese were also more likely to report everyday experiences of discrimination than Vietnamese: aOR = 1.48 [95% CI: 1.22–1.79].

Females were significantly more likely to report experiences of everyday discrimination compared to men, as were non-heterosexuals compared to heterosexuals: aOR = 1.24 [95% CI: 1.08–1.43] and aOR = 1.70 [95% CI: 1.21–2.39], respectively. Compared to participants who were 60 years or older, those who were younger than 30 years (aOR = 1.93 [95% CI: 1.46–2.56]), aged 30–39 (aOR = 1.60 [95% CI: 1.23–2.06]), and aged 40–49 (aOR = 1.36 [95% CI: 1.07–1.74]) were significantly more likely to experience everyday discrimination.

There were significant differences by region, with those living in Midwestern states significantly more likely to report experiences of everyday discrimination compared to those in Western states: aOR = 2.14 [95% CI: 1.60–2.86]. There were also significant differences for those who reported a severe change in their family income/employment compared to those who reported no change: aOR = 3.19 [95% CI: 2.10–4.85]. Self-reported health status was also a significant correlate of experience of everyday discrimination. Those who had lower self-reported health were significantly more likely to report experiences of everyday discrimination (a0Rs ranged from 1.38 to 2.02). Length of SIP and perceived severity were also significantly associated with experiences of everyday discrimination. LEP was marginally significant, while marital status, employment status, and education were not significantly associated with experiences of everyday discrimination in the final model.

## 4. Discussion

This paper presents one of the first national studies that examined the prevalence of, and factors associated with discrimination experiences attributable to being AAPI among nearly 5000 AAPIs during the COVID-19 pandemic. Overall, an alarming proportion of participants (60.7%) reported discrimination experiences from October 2020–February 2021. In a survey administered in the early months of the COVID-19 outbreak with a sample of 410 ASAs, there was a 29% increase in discrimination experiences as a result of the pandemic [40]. Another national survey reported that 39% of U.S. adults agreed it was more common for people to express racist or racially insensitive views about ASAs compared to before the pandemic [41]. Our finding is also comparable to a few studies conducted during the height of the pandemic from other countries that have also reported an increased prevalence of discrimination experiences by Asians and persons of Asian background. In a crowdsourcing survey with over 35,000 Canadians, 64.4% of Korean Canadians and 59.6% of Chinese Canadians reported having experienced discrimination or having been treated unfairly by others during the pandemic [42]. As high as 84.5% of Asian Australians (n = 334) of the 3043 adult Australians reported experiencing some form of discrimination due to their ethnic origin [43]. The ongoing surveillance of discrimination experiences against ASAs is critical as these negative experiences are directly linked to poor health outcomes [44].

This study identified several sociodemographic factors that suggested some groups were more vulnerable than others in experiencing racial discrimination. These included self-reporting as female, non-heterosexual, younger age, and those living in the non-Western region of the U.S. Multiple reasons that were not examined in this study may potentially explain these results. For example, historically, ASA women had to contend with the intersectional experiences with sexism/sexualized racism [45]. The pandemic may have exacerbated discriminatory experiences in ASAs who identify as non-heterosexual since sexual/gender minorities experience discrimination on multiple levels including access to health care [46]. Younger respondents (<30 years) being more likely to report discrimination compared to older respondents (40–49; 50–59 years) may be related to older respondents being more desensitized to microaggressions/bias/discrimination from past negative experiences [47]. Additionally, given that a majority of the survey was administered online, only 21% of the respondents were >60 years of age and many were strictly adherent to SIP recommendations due to the high rates of COVID-19-related morbidity/mortality among older adults. Nonetheless, the age differences could not be explained by the variables examined in this study.

Another important finding was that poorer self-reported health was significantly associated with discrimination, which is consistent with prior discrimination research in AAPIs [44]. While the cross-sectional nature of this study could not establish causal associations, this finding suggested that those who were poorer in health were more likely to have experienced discrimination that might in turn, further worsen one’s health.

This study is among the first that revealed the association between COVID-19 specific contexts and discrimination experience that were attributable to being an AAPI, independent of individuals’ background or characteristics. For example, having an SIP order of 1 month or longer increased one’s likelihood to report discrimination experiences. However, it is unknown how adherent participants were to staying at home; nonetheless, SIP decreased in-person contact, which might have decreased exposure to discrimination [48]. Of note, for some participants, these surveys were collected during SIP orders, therefore it is unknown how much more discrimination would have been reported if no restrictions were in place (e.g., indoor activities, travel, etc.).

Participants who perceived a similar or higher severity of COVID-19 (vs. other places) where they lived were more likely to report discrimination. It is unclear whether such association could be related to anti-AAPI hate incidents. Participants who reported negative impacts on family income/employment due to COVID-19 were also more likely to have reported discrimination; however, it was unclear whether such experience could be explained by heightened needs to look for a job or to increase income.

Although racism and discriminatory acts against ASAs are not new (e.g., the Chinese Exclusion Act in 1882 that barred immigration solely based on race), the recent pandemic has exacerbated the xenophobia that exists not only against Chinese Americans, but other ASAs [49]. The perpetual-foreigner and yellow-peril stereotypes against ASAs are once again brought to the forefront as they were in 2003 during the SARS outbreak [50]. When there appear to be physical/financial threats brought on by a particular event/group, that group is targeted [51], as ASAs are today during the pandemic.

The impacts of discrimination experiences may have far-reaching consequences. A Canadian study showed that participants who experienced discrimination are less likely to have trust and confidence in institutions such as the government [42]. In an Australian survey of over 2000 participants, those who had experienced discrimination during the pandemic had lower levels of trust in institutions and a significant proportion of participants who haven’t experienced discrimination indicated that they avoided going to certain places or being in situations due to an anticipation of discrimination [48]. The anticipation of discrimination may be linked to media reports of a rise in anti-Asian racism and xenophobia globally [1] (the United States is no exception [2]) and on social media spaces [52,53,54] that perpetuate stereotypes and reinforce prejudice towards individuals of Asian background. Taken together, discrimination experiences can have adverse manifestations, for example, as it relates to the COVID-19 vaccine uptake. A recent national survey demonstrated a link between racial discrimination and increased vaccine hesitancy in a sample of 2650 Americans [55]. Prior studies using COMPASS data showed that AAPIs’ willingness to receive and concerns related to the COVID-19 vaccine varied across cultural groups [56,57]. These findings suggest that discrimination may be a key factor in explaining the disproportionately lower COVID-19 vaccine uptake, particularly among individuals/groups who historically, and in light of COVID-19-related anti-Asian sentiments, have already experienced discrimination or anticipate facing discrimination, and consequently, thwarting pandemic response.

COMPASS is one of the few national COVID-19 surveys for AAPIs, and the first to report on discrimination experience during the COVID-19 pandemic due to being an AAPI. Another strength of this study is that it was offered in multiple languages, which is important since research has shown that language access has important implications for health, health-care access, and research participation [58,59,60]. Moreover, because language access is important, future research should strongly consider adding more AAPI languages to the survey in order to remove the participation barrier due to language. Not only was the survey available online, but respondents were also able to complete the survey in several languages by phone with staff, and, where available, in person. The EDS is based on self-report and subjective feelings. However, in research on discrimination, perceptions of discrimination can be just as informative and harmful as objective measures of discrimination [61]. Williams and others have asserted that regardless of verified accuracy of reports of perceived discrimination, these experiences can lead to significantly greater stress, which unquestionably has severe consequences for health [23]. Even though the reports of discrimination for this study was high (60.7% overall), it is plausible that participants under-reported their discrimination experiences. Some participants may not have wanted to share their experience with discrimination for reasons such as wanting to provide a positive portrayal of themselves [62]. Future research may consider adding a social-desirability-bias scale to account for this potential bias. Additionally, response choices (e.g., “rarely” vs. “sometimes”) may be interpreted differently by different respondents. Furthermore, the COMPASS findings (as were previous surveys [40,41]) are cross-sectional so future research should consider a longitudinal design to investigate discrimination experiences over time and long-term impacts to AAPIs’ health.

## 5. Conclusions

Racist rhetoric and belief systems have plagued AAPIs for generations and have been linked to poor mental and physical health [63]. This study answers a call to action to provide empirical evidence on discrimination among AAPIs that has been deficient during the COVID-19 pandemic. The results revealed that discrimination experienced during the COVID-19 pandemic due to being AAPIs is a public health priority in the U.S. Considering the larger sociopolitical landscape of anti-AAPI hate in the U.S., there is a dire need for programs and policies to combat this. In a statement from the White House Initiative on AAPIs in May 2021, a whole-of-government agenda outlined plans for a federal response ranging from collecting disaggregated data on AAPIs, expanding language access, building a workforce to relieve poverty, addressing bullying, harassment, and bullying in schools, etc. [64]. With the COVID-19 Hate Crimes Act on 20 May 2021 [65], federal lawmakers enacted hate-crime legislation; however, it is upon state/local/tribal law enforcement agencies to create online reporting processes, collect data on disaggregated minorities, and expand education with guidance from the Department of Justice. Other efforts must include having greater political AAPI federal/state/local leadership representation in order to create relevant legislation. In addition, our nation’s educational system can have an impact on dismantling the deep roots of our racism/discrimination/xenophobia by requiring inclusion of AAPIs’ history. Our research findings may inform anti-AAPI discrimination policies/programs, particularly as it relates to the “model minority” narrative that states that AAPIs are without problems such as discrimination.

## Figures and Tables

**Table 1:** COMPASS study sample characteristics (N = 4971).

	N	%
Cultural Group		
Asian Indian	287	5.8
Ethnic Chinese ^1^	1685	33.9
Filipino	173	3.5
Hmong	110	2.2
Japanese	220	4.4
Korean	1119	22.5
Mixed ^2^	219	4.4
NHPI	116	2.3
Other	28	0.6
Other South-East Asian	36	0.6
Other South Asian	20	0.4
Vietnamese	958	19.3
**Sex**		
Female	3186	64.1
Male	1744	35.1
Other/Decline to State ^3^	41	0.8
**Sexual Orientation**		
Heterosexual	4533	91.2
Not Heterosexual	224	4.5
Decline to State	214	4.3
**Age (in years)**	45.2 (16.4) ^4^; Range: 18–97	
<30	1107	22.3
30–39	872	17.5
40–49	909	18.3
50–59	590	20.6
>60	1057	21.3
**Nativity**		
US-born	1753	35.3
Foreign-born	3152	63.4
**Years in U.S.**	25.6 (15.1) ^4^; Range: 0–82	
Don’t know	65	1.3
**Limited English Proficiency (LEP) ^5^**		
Yes	1109	22.3
No	3862	77.7
**Caregiver**		
Yes	1197	24.1
No	3774	75.9
**Marital Status**		
Single	1380	27.8
Married/Living with Partner	3219	64.8
Separated/Divorced/Widowed	333	6.7
Declined	39	0.8
**Employment Status**		
Full-time	2313	46.5
Part-time	842	16.9
Homemaker	417	8.4
Unemployed	515	10.4
Retired	559	11.3
Other/Decline to state	325	6.5
**Education**		
High school or less	779	15.9
Some college or technical school	582	11.9
Bachelor’s degree	1803	36.9
Master’s degree or higher	1724	35.3
**Annual Household Income ($)**		
≤25,000	805	16.2
>25,000–75,000	1373	27.6
>75,000–150,000	1248	25.1
>150,000	970	19.5
Decline to state	575	11.6
**Census Region**		
West	3219	64.8
Midwest	431	8.7
Northeast	607	12.2
South	713	14.3
**Self-reported Health** **Quintiles (range of health score)**	77.9 (15.2) ^4^; Range: 0–100	
Q1 (0–69)	823	17.5
Q2 (70–78)	1073	22.8
Q3 (79–83)	934	19.9
Q4 (84–89)	778	16.6
Q5 (90–100)	1092	23.2
**Length of SIP ^6^ Order**		
No order	327	6.6
<1 month	270	5.5
1 to <2 months	563	11.4
2 to <3 months	570	11.5
3 months or longer	2783	56.2
Do not know	438	8.9
**The Severity of COVID-19 Where You Live**		
A lot less	415	8.4
Somewhat less	862	17.4
About the same	1098	22.2
Somewhat more	1475	29.8
A lot more	1098	22.2
**COVID-19 Effect on Family Income/Employment**		
No Change	2034	41.1
Mild	1520	30.7
Moderate	1236	25
Severe	161	3.2

^1^ Ethnic Chinese includes mainland Chinese, Hongkonger, Taiwanese, and Huaren. ^2^ Native Hawaiians/Pacific Islanders. ^3^ Other: n = 7; Decline: n = 6. ^4^ Mean (SD). ^5^ Self-reported English proficiency categorized as limited (“yes”) if speaking or reading or writing English indicated as “some,” “a little” or “not at all”. ^6^ SIP: Shelter-in-Place.

**Table 2:** Experiences of discrimination during the COVID-19 pandemic due to being Asian, Asian American or Pacific Islander by cultural group: N (%).

	All	Asian Indian (n = 287)	Ethnic Chinese ^1^ (n = 1685)	Filipino (N = 173)	Hmong (n = 110)	Japanese (n = 220)	Korean (n = 1119)	NHPI ^2^ (n = 116)	Vietnamese (n = 958)	Other/Mixed (n = 303)
EDS_Overall: Any Discrimination Experience During COVID-19 Due to Being Asian American/Pacific Islander										
Yes	3018 (60.7)	119 (41.5)	1090 (64.7)	106 (61.3)	88 (80.0)	127 (57.7)	718 (64.2)	47 (40.5)	534 (55.7)	189 (62.4)
No	1953 (39.3)	168 (58.5)	595 (35.3)	67 (38.7)	22 (20.0)	93 (42.3)	401 (35.8)	69 (59.5)	424 (44.3)	114 (37.6)
EDS_1: you been treated with less respect than other people...										
Yes	2562 (51.5)	96 (32.7)	961 (57.0)	79 (45.7)	76 (69.1)	107 (48.6)	607 (54.2)	25 (30.2)	438 (45.7)	165 (54.5)
No	2409 (48.5)	193 (67.3)	724 (43.0)	94 (54.3)	34 (30.9)	113 (51.4)	512 (45.8)	91 (69.8)	520 (54.3)	138 (45.5)
EDS_2: you been treated unfairly at restaurants or stores...										
Yes	1685 (33.9)	67 (23.3)	649 (48.5)	52 (30.1)	60 (54.5)	65 (29.5)	349 (31.2)	26 (22.4)	303 (31.6)	114 (37.6)
No	3286 (66.1)	220 (76.7)	1036 (61.5)	121 (69.9)	50 (45.5)	155 (70.5)	770 (68.8)	90 (77.6)	655 (68.4)	189 (62.4)
EDS_3: people criticized your accent or the way you speak...										
Yes	1344 (27.0)	73 (35.4)	488 (39.0)	41 (23.7)	43 (39.1)	33 (15.0)	318 (28.4)	22 (19.0)	244 (25.5)	82 (27.1)
No	3627 (73.0)	214 (74.6)	1197 (71.0)	132 (76.3)	67 (60.9)	187 (85.0)	801 (71.6)	94 (81.0)	714 (74.5)	221 (72.9)
EDS_4: people acted as if they think you are not smart...										
Yes	1278 (25.7)	60 (20.9)	486 (28.8)	43 (24.9)	58 (57.7)	34 (15.4)	257 (23.0)	31 (26.7)	234 (24.4)	75 (24.7)
No	3693 (74.3)	227 (79.1)	1199 (71.2)	130 (75.1)	52 (42.3)	186 (84.6)	862 (77.0)	85 (73.3)	724 (75.6)	228 (75.3)
EDS_5: people acted as if they are afraid of you...										
Yes	1781 (35.8)	55 (19.2)	705 (41.8)	50 (28.9)	73 (66.4)	72 (32.7)	359 (32.1)	31 (26.7)	313 (32.7)	123 (40.6)
No	3190 (64.2)	232 (80.8)	980 (58.2)	123 (71.1)	37 (33.6)	148 (67.3)	760 (67.9)	85 (73.3)	645 (67.3)	180 (59.4)
EDS_6: people acted as if they think you are dishonest...										
Yes	1113 (22.4)	53 (18.5)	447 (26.5)	33 (19.1)	47 (42.7)	38 (17.3)	184 (16.4)	25 (21.5)	209 (21.8)	77 (25.4)
No	3858 (77.6)	234 (81.5)	1238 (73.5)	140 (80.9)	63 (57.3)	182 (82.7)	935 (83.6)	91 (78.5)	749 (78.2)	226 (74.6)
EDS_7: people acted as if they’re better than you are…										
Yes	1878 (37.8)	84 (29.3)	699 (41.5)	75 (43.3)	69 (62.7)	75 (34.1)	409 (36.5)	34 (29.3)	309 (32.2)	124 (40.9)
No	3093 (62.2)	203 (70.7)	986 (58.5)	98 (56.7)	41 (37.3)	145 (65.9)	710 (63.5)	82 (70.7)	649 (67.8)	179 (59.1)
EDS_8: you been threatened or harassed…										
Yes	1282 (25.8)	53 (18.5)	562 (33.3)	41 (23.7)	49 (44.5)	50 (22.7)	231 (20.6)	18 (15.5)	195 (20.3)	83 (27.4)
No	3689 (74.2)	234 (81.5)	1123 (66.7)	132 (76.3)	61 (55.5)	170 (77.3)	888 (79.4)	98 (84.5)	763 (79.7)	220 (72.6)

^1^ Ethnic Chinese includes mainland Chinese, Hongkonger, Taiwanese, and Huaren. ^2^ Native Hawaiians/Pacific Islanders.

**Table 3:** Experiences of discrimination during the COVID-19 pandemic due to being Asian, Asian American or Pacific Islander by cultural group: N (%).

Variables	Experience of Discrimination			
	Yes N = 3018 (%)	No N = 1953 (%)	Crude OR (95% CI)	Adjusted OR (95% CI)
**Cultural Group**				
Asian Indian	119 (41.5)	168 (58.5)	Reference	Reference
Ethnic Chinese ^1^	1090 (64.7)	595 (35.3)	**2.59 (2.00–3.34)**	**2.38 (1.79–3.17)**
Filipino	106 (61.3)	67 (38.7)	**2.23 (1.52–3.29)**	**1.81 (1.18–2.78)**
Hmong	88 (80.0)	22 (20.0)	**5.65 (3.35–9.53)**	**2.09 (1.10–3.97)**
Japanese	127 (57.7)	93 (42.3)	**1.93 (1.35–2.75)**	**2.21 (1.49–3.28)**
Korean	718 (64.2)	401 (35.8)	**2.53 (1.94–3.29)**	**2.04 (1.50–2.78)**
NHPI ^2^	47 (40.5)	69 (59.5)	0.96 (0.62–1.49)	1.06 (0.64–1.76)
Vietnamese	534 (55.7)	424 (44.3)	**1.78 (1.36–2.32)**	**1.61 (1.18–2.20)**
Other/Mixed	189 (62.4)	114 (37.6)	**2.34 (1.68–3.26)**	**1.78 (1.23–2.57)**
**Sex**				
Female	1976 (62.0)	1210 (38.0)	**1.17 (1.04–1.32)**	**1.24 (1.08–1.43)**
Male	1015 (58.2)	729 (41.8)	Reference	Reference
Other/Decline to State	27 (65.9)	14 (34.1)	1.39 (0.72–2.66)	1.16 (0.48–2.85)
**Sexual Orientation**				
Heterosexual	2716 (59.9)	1817 (40.1)	Reference	Reference
Not Heterosexual	166 (74.1)	78 (25.9)	**1.91 (1.41–2.60)**	**1.70 (1.21–2.39)**
Decline to State	136 (63.6)	78 (36.4)	1.17 (0.88–1.55)	**1.45 (1.02–2.06)**
**Age (in years)**				
<30	773 (69.8)	334 (30.2)	**2.48 (2.08–2.96)**	**1.93 (1.46–2.56)**
30–39	579 (66.4)	293 (33.6)	**2.12 (1.76–2.55)**	**1.60 (1.23–2.06)**
40–49	566 (62.3)	343 (37.7)	**1.77 (1.48–2.12)**	**1.36 (1.07–1.74)**
50–59	590 (57.5)	436 (42.5)	**1.45 (1.22–1.73)**	1.22 (0.97–1.53)
>60	510 (48.2)	547 (51.8)	Reference	Reference
**Nativity**				
US-born	1104 (63.0)	649 (37.0)	**1.16 (1.03–1.31)**	NA
Foreign-born	1876 (59.5)	1276 (40.5)	Reference	
**LEP ^3^**				
Yes	626 (56.6)	483 (43.5)	Reference	Reference
No	2392 (61.9)	1470 (38.1)	**1.25 (1.10–1.43)**	1.21 (1.00–1.47)
**Caring**				
Yes	736 (61.5)	461 (38.5)	1.04 (0.91–1.19)	NA
No	2282 (60.5)	1492 (39.5)	Reference	
**Marital Status**				
Single	907 (65.7)	473 (34.3)	**1.32 (1.16–1.51)**	0.89 (0.74–1.08)
Married/Living with Partner	1907 (59.3)	1312 (40.8)	Reference	Reference
Separated/Divorced/Widowed	178 (53.5)	155 (46.5)	**0.79 (0.63–0.99)**	1.06 (0.82–1.38)
Declined	26 (66.7)	13 (33.3)	1.04 (0.53–2.05)	1.16 (0.47–2.85)
**Employment Status**				
Full-time	1451 (62.7)	862 (37.3)	**2.04 (1.69–2.45)**	1.27 (0.97–1.66)
Part-time	533 (63.3)	309 (36.7)	**2.09 (1.68–2.59)**	1.15 (0.87–1.52)
Homemaker	253 (60.7)	164 (39.3)	**1.87 (1.44–2.41)**	1.17 (0.84–1.63)
Unemployed	328 (63.7)	187 (36.3)	**2.12 (1.66–2.71)**	1.10 (0.79–1.51)
Retired	253 (45.3)	306 (54.7)	Reference	Reference
Other/Decline to state	200 (61.5)	125 (38.5)	0.95 (0.75–1.21)	0.93 (0.65–1.34)
**Education**				
High school or less	436 (56.0)	343 (44.0)	Reference	Reference
Some college/technical school	380 (65.3)	202 (34.7)	**1.48 (1.19–1.85)**	1.16 (0.90–1.49)
Bachelor’s degree	1090 (60.5)	713 (39.5)	**1.20 (1.02–1.43)**	0.95 (0.77–1.17)
Master’s degree or higher	1064 (61.7)	660 (38.3)	**1.27 (1.07–1.51)**	1.19 (0.94–1.50)
**Annual Household Income ($)**				
≤25,000	451 (56.0)	354 (44.0)	Reference	N/A
>25,000–75,000	881 (64.2)	492 (35.8)	**1.41 (1.18–1.68)**	
>75,000–150,000	823 (66.0)	425 (34.0)	**1.52 (1.27–1.82)**	
>150,000	548 (56.5)	422 (43.5)	1.02 (0.94–1.23)	
Decline to state	315 (54.8)	260 (45.2)	0.95 (0.77–1.18)	
**Census Region**				
West	1825 (56.7)	1394 (43.3)	Reference	Reference
Midwest	328 (76.1)	103 (23.9)	**2.43 (1.93–3.07)**	**2.14 (1.60–2.86)**
Northeast	387 (63.8)	220 (36.2)	**1.34 (1.12–1.61)**	**1.32 (1.07–1.63)**
South	478 (67.0)	235 (33.0)	**1.55 (1.31–1.84)**	**1.55 (1.27–1.89)**
**Self-reported Health Quintiles**				
Q1 (lowest)	568 (69.0)	255 (31.0)	**2.13 (1.76–2.58)**	**2.02 (1.64–2.47)**
Q2	692 (64.5)	381 (35.5)	**1.74 (1.46–2.07)**	**1.59 (1.32–1.92)**
Q3	570 (61.0)	364 (39.0)	**1.50 (1.26–1.79)**	**1.43 (1.18–1.72)**
Q4	466 (59.9)	312 (40.1)	**1.43 (1.19–1.72)**	**1.38 (1.13–1.69)**
Q5 (highest)	558 (51.1)	534 (48.9)	Reference	Reference
**Length of SIP ^4^ Order**				
No order	166 (50.8)	161 (49.2)	Reference	Reference
<1 month	161 (59.6)	109 (40.4)	**1.59 (1.21–2.10)**	1.20 (0.84–1.73)
1 to <2 months	350 (62.2)	213 (37.8)	**2.24 (1.69–2.97)**	**1.43 (1.04–1.96)**
2 to <3 months	398 (69.8)	172 (30.2)	**1.44 (1.15–1.82)**	**1.85 (1.34–2.57)**
3 months or longer	1665 (59.8)	1118 (40.2)	**1.43 (1.03–1.98)**	**1.43 (1.09–1.89)**
Do not know	268 (61.2)	170 (38.8)	**1.53 (1.14–2.04)**	1.30 (0.93–1.82)
**The severity of COVID-19 where you live**				
A lot less	199 (47.9)	216 (52.1)	Reference	Reference
Somewhat less	487 (56.5)	375 (43.5)	**1.41 (1.11–1.78)**	1.15 (0.89–1.50)
About the same	701 (63.8)	397 (36.2)	**1.92 (1.53–2.41)**	**1.52 (1.18–1.97)**
Somewhat more	942 (63.9)	533 (36.1)	**1.92 (1.54–2.39)**	**1.52 (1.18–1.94)**
A lot more	676 (61.6)	422 (38.4)	**1.74 (1.39–2.18)**	**1.37 (1.06–1.77)**
**COVID-19 Effect on Family Income/Employment**				
No Change	1078 (53.0)	956 (47.0)	Reference	Reference
Mild	970 (63.8)	550 (36.2)	**1.56 (1.37–1.79)**	**1.46 (1.25–1.69)**
Moderate	838 (67.8)	398 (32.2)	**1.87 (1.61–2.16)**	**1.95 (1.64–2.32)**
Severe	123 (76.4)	38 (23.6)	**2.87 (1.98–4.17)**	**3.19 (2.10–4.85)**

^1^ Ethnic Chinese includes mainland Chinese, Hongkonger, Taiwanese, and Huaren. ^2^ Native Hawaiians/Pacific Islanders. ^3^ Self-reported English proficiency categorized as limited (“yes”) if speaking or reading or writing English indicated as “some,” “a little” or “not at all”. ^4^ SIP: Shelter-in-Place. Odds ratios that were statistically significant from the logistic regression models were bolded.

## Data Availability

We will follow the National Institutes of Health data sharing policy, https://grants.nih.gov/grants/policy/data_sharing/ (accessed on 30 November 2021). To request data from this study, please contact Van Ta Park (van.park@ucsf.edu).
